# In Vivo and In Vitro Characteristics of Radiolabeled Vesamicol Analogs as the Vesicular Acetylcholine Transporter Imaging Agents

**DOI:** 10.1155/2018/4535476

**Published:** 2018-06-13

**Authors:** Kazuma Ogawa, Kazuhiro Shiba

**Affiliations:** ^1^Kanazawa University, Graduate School of Pharmaceutical Sciences, Kakuma-machi, Kanazawa, Ishikawa 920-1192, Japan; ^2^Advanced Science Research Center, Kanazawa University, 13-1 Takara-machi, Kanazawa, Ishikawa 920-8640, Japan

## Abstract

The vesicular acetylcholine transporter (VAChT), a presynaptic cholinergic neuron marker, is a potential internal molecular target for the development of an imaging agent for early diagnosis of neurodegenerative disorders with cognitive decline such as Alzheimer's disease (AD). Since vesamicol has been reported to bind to VAChT with high affinity, many vesamicol analogs have been studied as VAChT imaging agents for the diagnosis of cholinergic neurodeficit disorder. However, because many vesamicol analogs, as well as vesamicol, bound to sigma receptors (*σ*_1_ and *σ*_2_) besides VAChT, almost all the vesamicol analogs have been shown to be unsuitable for clinical trials. In this report, the relationships between the chemical structure and the biological characteristics of these developed vesamicol analogs were investigated, especially the in vitro binding profile and the in vivo regional brain accumulation.

## 1. Introduction

**Figure 1:**
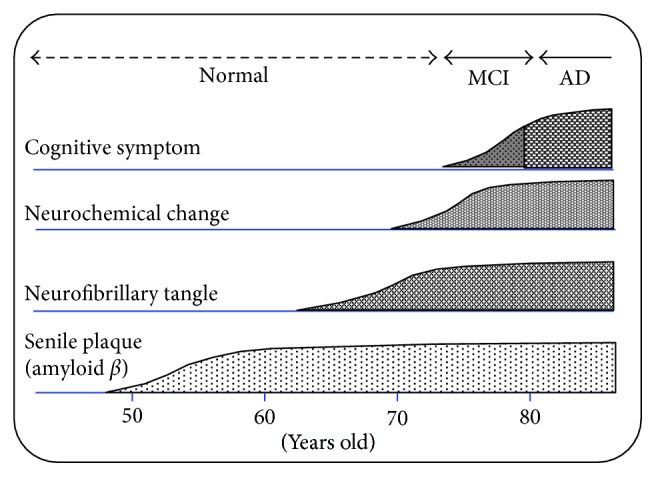
Process of the Alzheimer's disease onset.

**Figure 2:**
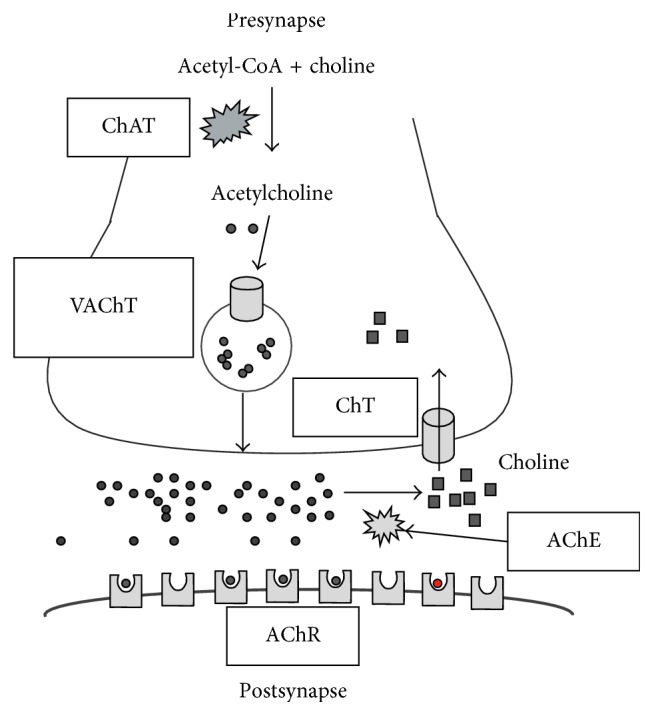
Schematic representation of a nerve terminal in acetylcholine neurotransmission. ChAT: choline acetyl transferase; VAChT: acetylcholine transporter; AChR: acetylcholine receptor; AChE: acetylcholine esterase; ChT: choline transporter.

Many clinical trials for early diagnosis of Alzheimer's disease by amyloid PET imaging have been reported [1–10]. Many researchers have reported that amyloid imaging is useful for early diagnosis of AD, but there have been many reports showing no significant association between the brain accumulation of amyloid imaging agents and the severity of dementia in AD [11–15]. Recently, there were many reports that tau imaging was useful for the diagnosis of the severity of dementia and early diagnosis of AD [16–20]. Evaluation of the diagnostic efficacy of tau imaging regarding AD will continue for several years. The onset of AD, which is a progressive neurological disease characterized by reduction in cognitive function and memory, is thought to be caused by a hypothesized amyloid cascade (Figure 1) [21]. Namely, (1) amyloid *β* (A*β*40 and A*β*42), which is produced by an abnormal cleavage of the amyloid precursor protein (APP) by *β*- and *γ*-secretase, is aggregated and accumulated extracellularly in cranial nerve cells. (2) Neurofibrillary tangles (NFTs) are formed by the accumulation of a tau protein phosphorylated excessively in the cytoplasm. (3) Nerve degeneration, neurologic function deficiency, and metabolism deficiency occur in the neuronal cell. (4) Neuronal cell death occurs, which causes the onset of AD. Abnormal accumulation of amyloid *β* based on the amyloid cascade supports the usefulness of amyloid imaging for early diagnosis of AD. On the contrary, studies of amyloid *β* immunotherapy showed that reduction of amyloid *β* plaques in patients with Alzheimer's disease did not prevent progressive neurodegeneration [22]. The amyloid *β* plaque is an antecedent marker of Alzheimer's disease [22], and amyloid imaging will not be useful to evaluate the therapeutic efficacy of AD treatment. Neuronal degeneration, neurologic function deficiency, and metabolic deficiency in the third step of the amyloid cascade are thought to be important internal molecular targets for the development of an imaging agent for the early diagnosis of neurodegenerative disorders with cognitive decline such as AD. Acetylcholine esterase inhibitors such as donepezil are commonly used for treatment of cognitive dysfunction in AD [23, 24]. The dysfunction of cholinergic neurons is associated with AD symptoms such as cognitive dysfunction, memory impairment, and learning disorders [25]. Presynaptic cholinergic function, such as loss of choline acetyl transferase (ChAT), the enzyme for synthesis of acetylcholine (ACh) from choline and acetyl-coenzyme A, and the vesicular acetylcholine transporter (VAChT), the transporter for the accumulation of acetylcholine (ACh) inside the synaptic vesicles, is changed in AD [26, 27]. Thus, the internal molecules in the cholinergic nerve system will be suitable as the cranial molecular target of an imaging agent for early diagnosis of AD. There are five main molecular targets: choline acetyl transferase (ChAT), vesicular acetylcholine transporter (VAChT), choline transporter (ChT), acetylcholine esterase (AChE), and postsynaptic receptors in the cholinergic synaptic terminal (Figure 2). A small molecule compound binding to ChAT with high affinity has not yet been found, and only hemicholinium-3 (HC-3) with a positive electric charge as a small molecule compound binding to ChT, the transporter for reuptake of choline (Ch) released by ACh hydrolysis in the synaptic cleft, has been found, which makes the development of ChAT and ChT imaging agents difficult. A reduction in AChE activity in AD patients was shown by PET imaging using [^11^C]MP4A and [^11^C]PMP [28–31]. However, these AChE imaging agents, a selective substrate for AChE, show low stability in the blood, and quantitative measurement is thought to be difficult. Changes in presynaptic cholinergic functions, such as ChAT and VAChT activity in AD, are thought to be more significant than changes in postsynaptic cholinergic functions, such as the cholinergic muscarinic receptors (mAChR) [26, 27, 32]. Therefore, VAChT is an excellent in vivo target substrate for the early diagnosis of AD. Many vesamicol analogs have been developed as potential VAChT imaging agents for PET or SPECT, since vesamicol (2-(4-phenylpiperidino)cyclohexanol) was reported to bind to VAChT [33, 34]. However, many of the reported vesamicol analogs were shown to be insufficient for use as VAChT imaging agents due to binding to sigma receptors (*σ*_1_ and *σ*_2_) or low accumulation in brain in vivo. Many vesamicol analogs were developed with the aim of improving VAChT affinity and decreasing the affinity for sigma receptors (*σ*_1_ and *σ*_2_).

In this report, the biological characteristics of these developed vesamicol analogs were investigated, especially the in vitro binding profile and the in vivo regional brain accumulation. PET ligands for VAChT had been reviewed by Giboureau previously [35]. We tried to compare in vitro and in vivo characteristics of many VAChT ligands including these PET ligands under the same conditions as much as possible.

## 2. VAChT Imaging Agent Based on Vesamicol Analogs

**Figure 3:**
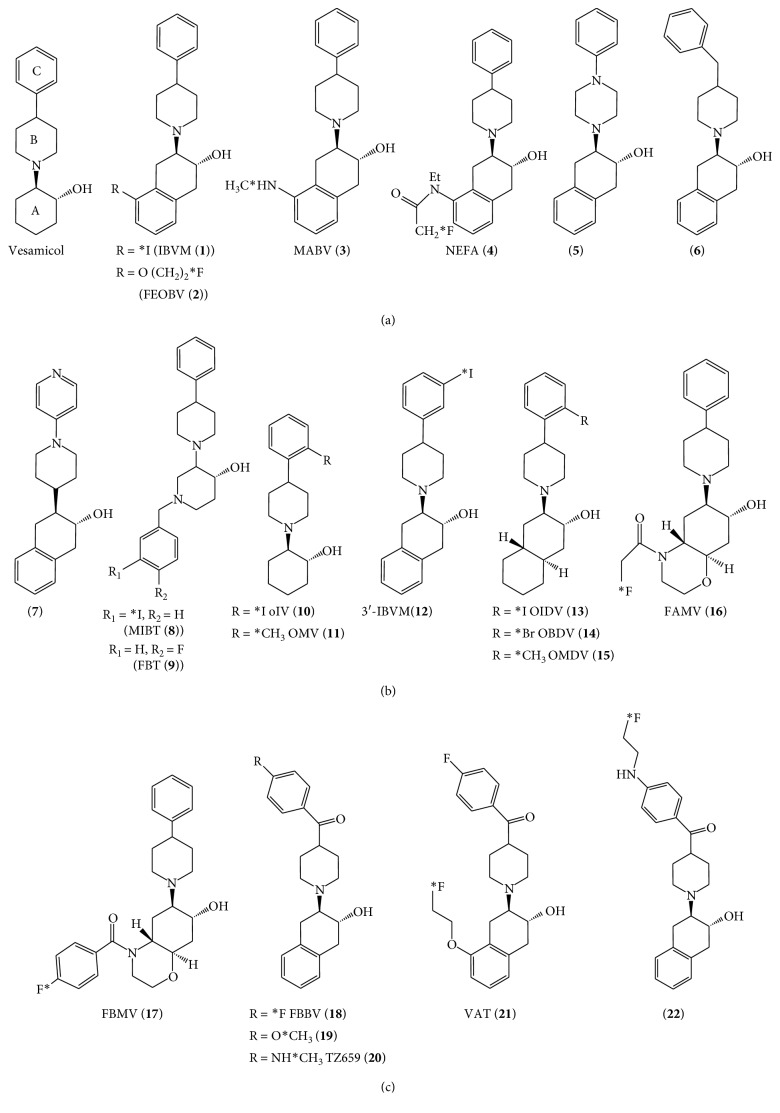
Molecular structures of vesamicol analogs. Benzovesamicol analogs: (**1**)–(**7**). Trozamicol analogs: (**8**) and (**9**). Vesamicol analogs with a radionuclide into the C ring: (**10**)–(**12**). Vesamicol analogs with alicyclic groups into the A ring: (**13**)–(**17**). Vesamicol analogs with a carbonyl group between the B ring and C ring: (**18**)–(**22**).

The molecular structures of VAChT imaging agents based on vesamicol analogs are shown in Figure 3.

### 2.1. Vesamicol Analogs Based on Benzovesamicol

#### 2.1.1. 5-Iodobenzovesamicol (IBVM) (**1**)

Rogers et al. investigated the binding affinity of 84 vesamicol analogs to VAChT in an in vitro binding assay [36] and reported benzovesamicol as one of the vesamicol analogs binding to VAChT with a high affinity. Benzovesamicol (BV) is the vesamicol analog with a benzene ring in ring A of the vesamicol skeleton (Figure 3). The affinity (IC_50_ = 50 nM) of the racemate of BV to VAChT is similar to that (IC_50_ = 40 nM) of the racemate of vesamicol in the in vitro binding assay [36]. (−)-[^123^I]-5-iodobenzovesamicol ((−)-[^123^I]IBVM) (**1**), which has an iodine-123-labeled group at the 5th position of the benzene ring in ring A, was first developed as a potential VAChT imaging agent. However, the affinity of IBVM to VAChT has not been reported [37, 38], and the accumulation of (−)-[^123^I]IBVM (**1**) in the rodent brain may be insufficient for in vivo brain imaging for SPECT, considering the radiation dose and spatial resolution. The accumulation of (−)-[^123^I]IBVM (**1**) in the cortex and striatum was 2.56 %ID/g and 6.26 %ID/g in mice 4 h after injection, respectively, and 0.30 %ID/g and 0.53 %ID/g in rats 2 h after injection, respectively [37]. The uptake of (−)-[^123^I]IBVM (**1**) in the striatum is much higher than that in the cortex, and the ratio of the striatum to cortex for (−)-[^123^I]IBVM (**1**) accumulation was 2.44 at 4 h after injection. Kuhl et al. extended to human use (−)-[^123^I]IBVM (**1**) for SPECT (single-photon emission computed tomography). Several researchers showed that a decrease in (−)-[^123^I]IBVM (**1**) binding was apparent in regional brain areas such as the temporal cortex, cingulate cortex, and parahippocampal-amygdaloid complex in AD [39, 40].

The human study using [^123^I]IBVM-SPECT showed that a significant decrease in [^123^I]IBVM (**1**) binding (47–62%) was apparent in AD subjects in the cingulate cortex and parahippocampal-amygdaloid complex [43].

#### 2.1.2. Fluoroethoxy-benzovesamicol (FEOBV) (**2**)

As a benzovesamicol analog for PET, [^18^F]fluoroethoxy-benzovesamicol ([^18^F]FEOBV (**2**)) was synthesized and investigated for regional brain distribution in mice by Mulholland et al. [44]. The binding affinity (Ki) of (−)-FEOBV (**2**) to VAChT and *σ*1 was 19.6 nM (PC12^A123.7^ cells, which express human VAChT) and 209 nM (rat brain), respectively [45]. However, in the in vitro binding assay, vesamicol as a reference was not assessed simultaneously. The accumulation of [^18^F]FEOBV (**2**) in the striatum in mice 5, 45, and 240 min after injection was 6.02, 8.09, and 7.29 %ID/g, and the accumulation of [^18^F]FEOBV (**2**) in the cortex in mice 5, 45, and 240 min after injection was 5.00, 4.65, and 2.91 %ID/g, respectively [46]. Thus, the mouse brain uptake of [^18^F]FEOBV (**2**) is insufficient for in vivo brain imaging with PET. The ratios of the striatum to cortex for [^18^F]FEOBV (**2**) accumulation were 1.20, 1.74, and 2.51 after 5, 45, and 240 min injection, respectively. The in vivo human studies demonstrated that [^18^F]FEOBV (**2**) is a reliable imaging tool for VAChT [47].

#### 2.1.3. Aminobenzovesamicol

*(−)-[* ^*11*^ *C]-Methylaminobenzovesamicol ((−)-[* ^*11*^ *C]MABV (* **3** *)) and (−)-[* ^*18*^ *F]-N*-*ethyl*-*N*-*fluoroacetamidobenzovesamicol ((−)-[*^*18*^*F]NEFA ( ***4***))*. As benzovesamicol analogs with the amino group, (−)-[^11^C]-methylaminobenzovesamicol ((−)-[^11^C]MABV (**3**)) [48] and (−)-[^18^F]-*N*-ethyl-*N*-fluoroacetamidobenzovesamicol ((−)-[^18^F]NEFA (**4**)) [49, 50] were reported. The accumulation of (−)-[^11^C]MABV (**3**) in the striatum, the cortex, the hippocampus, and the cerebellum in mice 45 min after injection was 8.10, 3.99, 3.58, and 1.05 %ID/g, although the binding affinity (Ki) of (−)-[^11^C]MABV (**3**) to VAChT and *σ*_1_ and *σ*_2_ receptors was not reported. The binding affinity (Ki) of (−)-[^18^F]NEFA (**4**) to VAChT was 0.32 nM and was about 3-fold higher than that of vesamicol. The binding affinity (Ki) of (−)-[^18^F]NEFA (**4**) to *σ*_1_ and *σ*_2_ receptors was not reported. The accumulation of (−)-[^18^F]NEFA (**4**) in the rat whole brain 60 min after injection was 0.35 %ID/g.

#### 2.1.4. Summary regarding Benzovesamicol Analogs

Although benzovesamicol (BV) has higher affinity and selectivity for VAChT than vesamicol, several benzovesamicol (BV) analogs were synthesized to improve VAChT affinity and to decrease the affinity for sigma receptors (*σ*_1_ and *σ*_2_). A BV analog with phenylpiperazine instead of phenylpiperidine in BV (**5**) [51, 52], a BV analog with benzylpiperidine instead of phenylpyridine (**6**), and a BV analog with pyridinyl piperidine instead of phenyl piperidine (**7**) showed a lower VAChT affinity than BV [53]. The affinity of **7** to the *σ*1 receptor increased.

### 2.2. Vesamicol Analogs Based on Trozamicol

#### 2.2.1. (+)-*m*-Iodobenzyltrozamicol (MIBT) (**8**)

The structure of *m*-iodobenzyltrozamicol (MIBT) (**8**) consists of a trozamicol skeleton, a compound with piperidine instead of cyclohexane in A ring, with an *m*-iodobenzyl group. The binding affinity of MIBT (**8**) to VAChT, *σ*_1_, and *σ*_2_ was 0.13 nM (*Torpedo californica*), 92 nM (Guinea pig membranes), and 190 nM (rat liver), respectively, and the binding affinity of vesamicol to VAChT, *σ*_1_, and *σ*_2_ was 2.0 nM (*Torpedo californica*), 26 nM (Guinea pig membranes), and 34 nM (rat liver), respectively [54]. (+)-MIBT (**8**) is the optical isomer of MIBT. The binding affinity of (+)-MIBT (**8**) to VAChT (Ki = 12.2 nM (rat brain)), *σ*1 (Ki = 8.4 nM (Guinea pig brain)), and *σ*2 (Ki = 40.4 nM (N18TG2 cell fused with septal neurons rat (*σ*_2_)) and the binding affinity of (−)-vesamicol to VAChT (Ki = 26.6 nM), *σ*_1_ (Ki = 37.6 nM), and *σ*_2_ (Ki = 42.3 nM) were reported by Custers et al. [55]. (+)-*m*-[^125^I]-iodobenzyltrozamicol ((+)-[^125^I]MIBT (**8**)) accumulation in the rat brain was 0.57, 0.36, 0.27, and 0.15 %ID/g at 5, 30, 60, and 180 min after injection, respectively. The ratio of the striatum to cortex for (+)-[^125^I]MIBT (**8**) accumulation was 1.73–2.34 at 180 min after injection. (+)-[^125^I]MIBT (**8**) showed rapid clearance from blood. Pretreatment of rats with vesamicol (1.01 *μ*mol/kg) decreased (+)-[^125^I]MIBT (**8**) accumulation in the rat brain by 40% [56]. Coadministration of (+)-[^125^I]MIBT (**8**) with haloperidol (0.5 *μ*mol/kg), a known sigma receptor ligand, decreased (+)-[^125^I]MIBT accumulation in the cortex and the cerebellum but increased (+)-[^125^I]MIBT (**8**) accumulation in the striatum. In further studies, preinjection of spiperone (2 mg/kg), a known dopamine D_2_ receptor ligand, increased (+)-[^125^I]MIBT (**8**) accumulation in the striatum. The increase of (+)-[^125^I]MIBT (**8**) accumulation in the striatum was thought to be caused by interaction between the dopaminergic nerve system and cholinergic nerve system [57]. A human SPECT study with (+)-[^123^I]MIBT (**8**) was not performed, but (+)-[^123^I]MIBT-SPECT study was performed using a female baboon [58, 59].

#### 2.2.2. (+)-*p*-Fluorobenzyltrozamicol (FBT) (**9**)

(+)-*p*-Fluorobenzyltrozamicol ((+)-FBT (**9**)) is a vesamicol analog having trozamicol with a *p*-fluorobenzyl group. The binding affinity (Ki) of (+)-FBT (**9**) to VAChT, *σ*_1_, and *σ*_2_ was 0.22 nM (*Torpedo californica*), 21.6 nM (Guinea pig brain), and 35.9 nM (rat liver), respectively, and the binding affinity (Ki) of (−)-vesamicol to VAChT, *σ*_1_, and *σ*_2_ was 1.0 nM (*Torpedo californica*), 25.8 nM (Guinea pig brain), and 34.5 nM (rat liver), respectively [60]. Giboureau et al. reported that (+)-FBT (**9**) showed a 100- to 160-fold selectivity for the VAChT/*σ*_1_/*σ*_2_ receptors, while (−)-vesamicol showed a range from 25- to 35-fold selectivity for the VAChT/*σ*_1_/*σ*_2_ receptors [35]. The (+)-[^18^F]FBT (**9**) accumulation in the rat brain was 0.82, 0.71, 0.59, 0.34, and 0.27 %ID/g at 5, 30, 60, 120, and 180 min after injection, respectively. The ratio of the striatum to cortex for (+)-[^18^F]FBT (**9**) accumulation increased with time after (+)-[^18^F]FBT (**9**) injection, and the striatum/cortex ratio was 1.89 at 180 min after injection [61]. Blocking studies using vesamicol, a VAChT ligand, or pentazocine, a selective *σ*_1_ ligand, had not been reported. However, coadministration of (+)-[^18^F]FBT (**9**) with haloperidol, a σ ligand and dopamine D_2_ receptor ligand, increased (+)-[^18^F]FBT (**9**) accumulation in the striatum. A radioactive metabolite of (+)-[^18^F]FBT (**9**) that did not cross the blood-brain barrier (BBB) was observed in PET studies using rhesus monkeys [60, 61]. A human PET study with (+)-[^18^F]FBT (**9**) has not been performed.

#### 2.2.3. Summary regarding Trozamicol Analogs

The binding affinity of each trozamicol analog was investigated by a different method of the in vitro binding assay. Furthermore, different tissue membranes were used in the binding assay for VAChT or sigma receptors (*σ*_1_ and *σ*_2_). It is difficult to compare the value of the VAChT affinity of each trozamicol analog with the value of sigma receptors (*σ*_1_ and *σ*_2_) affinity. Thus, to compare all vesamicol analogs, the value of the affinity of vesamicol to VAChT and sigma receptors (*σ*_1_ and *σ*_2_) was used as the standard value in each binding assay in this report. Namely, the comparison between these vesamicol analogs was expressed as the ratio of the affinity of vesamicol analogs to the affinity of vesamicol to VAChT, *σ*_1_, and *σ*_2_, which were obtained by the same in vitro binding assay method (Table 1). The affinity and selectivity of MIBT to VAChT was superior to that of FBT [57]. (+)-FBT (**9**) showed a higher affinity and selectivity for VAChT than (−)-FBT [60]. The accumulation of (+)-[^18^F]FBT (**9**) in the rat brain was higher than that of (+)-[^125^I]MIBT (**9**) [56, 72]. However, the accumulation of (+)-[^18^F]FBT (**9**) in the rat brain is lower than the expected brain accumulation based on the chemical structure of (+)-[^18^F]FBT (**9**) [72]. Considering the radiation dose and spatial resolution, the accumulation of (+)-[^18^F]FBT (**9**) in the rat brain is insufficient for in vivo PET brain imaging.

### 2.3. Vesamicol Analogs That Incorporated a Radionuclide into the C Ring of Vesamicol

#### 2.3.1. (−)-*o*-Iodovesamicol ((−)-oIV (**10**))

(−)-oIV (**10**) is a vesamicol analog having iodine at the *ortho*-position of the 4-phenylpiperidine moiety. It was reported that iodine at the *ortho*-position of the 4-phenylpiperidine moiety had no influence on affinity for VAChT and sigma receptors (*σ*_1_ and *σ*_2_) [62]. The binding affinity (Ki) of (−)-oIV (**10**) to VAChT, *σ*_1_, and *σ*_2_ was 15.0 nM (rat brain), 62.2 nM (rat brain), and 554 nM (rat liver), respectively, and the binding affinity (Ki) of (−)-vesamicol to VAChT, *σ*_1_, and *σ*_2_ was 13.0 nM (rat brain), 74.9 nM (rat brain), and 421 nM (rat liver), respectively. The accumulation of (−)-[^125^I]oIV (**10**) in the rat brain was 1.50, 1.27, and 0.76 %ID/g at 30, 60, and 120 min after injection, respectively. The ratio of the striatum to cortex for (−)-[^125^I]oIV (**10**) accumulation was 1.01 at 60 min after injection. The brain uptake of (−)-[^125^I]oIV (**10**) was decreased by 60% by coadministration of (−)-[^125^I]oIV (**10**) with vesamicol (0.5 *μ*mol/kg) [73, 74]. In the unilateral nucleus basalis magnocellularis- (NBM-) lesioned rat, the accumulation of (−)-[^125^I]oIV (**10**) in the ipsilateral cortex to the lesion was significantly lower than that in the contralateral cortex (about 17%) [74]. However, a human SPECT study with (−)-[^123^I]oIV (**10**) was not performed.

#### 2.3.2. (−)-*o*-Methylvesamicol ((−)-OMV (**11**))

(−)-OMV is a vesamicol analog having a methyl group at the *ortho*-position of the 4-phenylpiperidine moiety. The binding affinity (Ki) of (−)-OMV (**11**) to VAChT, *σ*_1_, and *σ*_2_ was 6.7 nM (rat brain), 33.7 nM (rat brain), and 266 nM (rat liver), respectively, and the binding affinity (Ki) of (−)-vesamicol to VAChT, *σ*_1_, and *σ*_2_ was 4.4 nM (rat brain), 73.8 nM (rat brain), and 346 nM (rat liver), respectively [63]. The (−)-[^11^C]OMV (**11**) accumulation in the rat brain was 1.13, 0.98, and 0.80 %ID/g at 5, 30, and 60 min after injection, respectively [75]. As reported by Giboureau et al. [35], (−)-[^11^C]OMV (**11**) was found to have the low brain uptake in primate (0.05% ID/mL) similar to (+)-[^18^F]FBT (0.05–0.06% ID/mL) [61]. However, in the unilateral MBM-lesioned rhesus monkeys, the accumulation of (−)-[^11^C]OMV (**11**) in the ipsilateral cortex to the lesion was significantly lower than that in the contralateral cortex. The reduction of the uptake of (−)-[^11^C]OMV (**11**) (27.5%) in the ipsilateral cortex to the lesion was greater than that of [^11^C]SA4503 (19.9%) [76]. A human SPECT study with (−)-[^11^C]OMV (**11**) was not performed.

#### 2.3.3. 3′-Iodobenzovesamicol (3′-IBVM (**12**))

3′-IBVM is a benzovesamicol analog with a 4-(3-iodophenyl)piperidine moiety [64]. The binding affinity (Ki) of 3′-IBVM (**12**) to VAChT, *σ*_1_, and *σ*_2_ was 2.0 nM (*Torpedo californica*), 70.3 nM (rat brain), and 43.6 nM (Guinea pig brain), respectively. The accumulation of 3′-[^125^I]IBVM (**12**) in the striatum and the cortex was 0.69 %ID/g and 0.57 %ID/g in rats 30 min after injection and 0.61 %ID/g and 0.38 %ID/g in the rat brain 60 min after injection, respectively. The ratio of the striatum to cortex for 3′-[^125^I]IBVM (**12**) accumulation was 1.21 and 1.61 at 30 and 60 min after injection, respectively. The accumulation of 3′-[^125^I]IBVM (**12**) in the striatum and the cortex was decreased by 28 and 70% by coadministration of 3′-[^125^I]IBVM (**12**) with haloperidol (0.5 *μ*mol/kg), respectively.

#### 2.3.4. Summary of Vesamicol Analogs That Incorporated Halogen or Methyl Group in 4-Phenylpiperidine

Iodine at the *ortho*-position of the 4-phenylpiperidine moiety of vesamicol has no influence on the affinity for VAChT and decreased the affinity for sigma receptors (*σ*_1_ and *σ*_2_) [62]. Iodine at the *meta*-position of the 4-phenylpiperidine moiety of benzovesamicol has no influence on the affinity for VAChT and sigma receptors (*σ*_1_ and *σ*_2_) [64]. An (−)-enantiomer of vesamicol analogs, which has a halogen or a methyl group in 4-phenylpiperidine and an (−)-enantiomer of vesamicol, showed a higher affinity for VAChT than an (+)-enantiomer [62, 63]. (−)-oIV (**10**) [74] and (−)-OMV (**11**) [75] showed a high brain uptake in rats. 3′-IBVM (**12**) [76] showed lower brain uptake in rats than (−)-oIV (**10**) and (−)-OMV (**11**).

### 2.4. Vesamicol Analogs That Incorporated Alicyclic Groups into the A Ring of Vesamicol

#### 2.4.1. (−)-*o*-Iodo-*trans*-decalinvesamicol ((−)-OIDV (**13**))

Decalinvesamicol (DV) showed the highest affinity for VAChT among 84 vesamicol analogs investigated by Rogers et al. (−)-OIDV (**13**) is a vesamicol analog having a decalin skeleton (as A ring) and a 4-(2-iodophenyl)piperidine moiety. The binding affinity (Ki) of (−)-OIDV (**13**) to VAChT, *σ*_1_, and *σ*_2_ was 22.1 nM (rat brain), 168 nM (rat brain), and 59.9 nM (rat liver), respectively. The ratio of the striatum to cortex for (−)-[^123^I]OIDV (**13**) accumulation was 1.20 at 60 min after injection. The rat brain uptake of (−)-[^123^I]OIDV (**13**) was decreased by 71–73% by coadministration of (−)-[^123^I]OIDV (**13**) with vesamicol (0.25 *μ*mol). (−)-[^123^I]OIDV (**13**) was distributed in characteristically VAChT-rich regions, such as the cortex, striatum, diagonal band, amygdaloid nucleus, and trigeminal and facial nucleus [65, 66, 77].

#### 2.4.2. *o*-Bromo-*trans*-decalinvesamicol ((−)-OBDV (**14**))

OBDV is a vesamicol analog having a decalin skeleton (as A ring) and a 4-(2-bromophenyl)piperidine moiety [67]. The binding affinity (Ki) of OBDV (**14**) to VAChT, *σ*_1_, and *σ*_2_ was 13.8 nM (rat brain), 150.7 nM (rat brain), and 137.5 nM (rat liver), respectively, and the binding affinity (Ki) of vesamicol to VAChT, *σ*_1_, and *σ*_2_ was 33.9 nM (rat brain), 22.1 nM (rat brain), and 86.7 nM (rat liver), respectively. The accumulation of [^77^Br]OBDV (**14**) in the striatum was 0.59 and 0.53 %ID/g in rats 30 and 60 min after injection, respectively, and the accumulation of [^77^Br]OBDV (**14**) in the cortex was 0.52 and 0.50 %ID/g in rats 30 and 60 min after injection, respectively. The ratio of the striatum to cortex for [^77^Br]OBDV (**14**) accumulation was 1.13 at 30 min after injection. The rat brain uptake of [^77^Br]OBDV (**14**) was remarkably decreased by 60% by coadministration of [^77^Br]OBDV (**14**) with vesamicol (0.25 *μ*mol). On the contrary, coadministration of (+)-pentazocine or (+)-3-PPP had no significant influence on the accumulation of [^77^Br]OBDV (**14**) in the rat brain.

#### 2.4.3. *o*-Methyl-*trans*-decalinvesamicol (OMDV (**15**))

OMDV is a vesamicol analog having a decalin skeleton (as A ring) and a 4-(2-methylphenyl)piperidine moiety [68]. The binding affinity (Ki) of OMDV (**15**) to VAChT, *σ*_1_, and *σ*_2_ was 11.9 nM (rat brain), 70.3 nM (rat brain), and 43.6 nM (rat liver), respectively, and the binding affinity (Ki) of vesamicol to VAChT, *σ*_1_, and *σ*_2_ was 21.1 nM (rat brain), 20.8 nM (rat brain), and 139 nM (rat liver), respectively. The accumulation of [^11^C]OMDV (**15**) in the cerebrum was 0.65 and 0.44 %ID/g in the rat brain 30 and 60 min after injection, respectively. The ratio of the striatum to cortex for [^11^C]OMDV (**15**) accumulation was 1.11 at 30 min after injection. A PET/CT study using [^11^C]OMDV (**15**) showed that the accumulation of [^11^C]OMDV (**15**) in the rat brain was markedly decreased by 60% by coadministration of 0.25 *μ*mol of vesamicol.

#### 2.4.4. Morpholinovesamicol Analogs

*2-[* ^*18*^ *F]fluoroacetyl morpholinovesamicol ([* ^*18*^ *F]FAMV (* **16** *))* [69] *and 4-[*^*18*^*F]fluorobenzoyl morpholinovesamicol ([*^*18*^*F]FBMV ( ***17***))* [70]. The binding affinity (Ki) of FAMV (**16**) to VAChT and *σ* receptors was 39.9 nM (PC12^A123.7^ cells, which express rat VAChT) and >1500 nM (rat liver), respectively. FAMV (**16**) showed about 30-fold lower affinity than vesamicol. The accumulation of [^18^F]FAMV (**16**) in the rat whole brain 60 min after injection was 0.35 %ID/g. The binding affinity (Ki) of FBMV (**17**) to VAChT and σ receptors was 27.5 nM (PC12^A123.7^ cells, which express rat VAChT) and >3000 nM (rat liver), respectively. FBMV (**17**) showed about 2.4-fold lower affinity than (−)-vesamicol. FBMV (**17**) showed a higher selectivity to VAChT affinity than (−)-vesamicol because FBMV (**17**) showed lower affinity for σ receptors than (−)-vesamicol. The accumulation of [^18^F]FBMV (**17**) in the rat whole brain 60 min after injection was 0.1 %ID/g.

#### 2.4.5. Summary of Vesamicol Analogs That Incorporated Alicyclic Groups into the A Ring of Vesamicol

Decalinvesamicol ((−)-OIDV (**13**) [66], OBDV (**14**) [67], and OMDV (**15**)) [68] showed a higher affinity to VAChT than morpholinovesamicol analogs (FAMV (**16**) and FBMV (**17**)). On the contrary, morpholinovesamicol analogs showed a higher selectivity to VAChT affinity than decalinvesamicol. The accumulation of radiolabeled decalinvesamicol analogs in the rat brain was higher than that of [^18^F]morpholinovesamicol analogs. However, radiolabeled decalinvesamicol analogs showed a lower brain uptake in rats than (−)-oIV (**10**) and (−)-OMV (**11**). The ratio of the striatum to cortex for [^18^F]morpholinovesamicol analogs (3.4–4.5) accumulation was higher than that of radiolabeled decalinvesamicol analogs (1.1–1.2).

### 2.5. Vesamicol Analogs That Incorporated a Carbonyl Group between the B Ring and C Ring of Benzovesamicol

#### 2.5.1. (−)-*trans*-2-Hydroxy-3-(4-(4-fluorobenzoyl)piperidino)tetralin ((−)-FBBV (**18**))

The affinity (Ki) of (−)-FBBV (**18**) to VAChT, *σ*_1_, and *σ*_2_ was 4.1 nM, 658.6 nM, and 319.23 nM, respectively [71, 78]. (−)-FBBV (**18**) displayed a 2-fold lower affinity for VAChT than vesamicol (Ki = 2.0 nM); furthermore, (−)-FBBV (**18**) displayed significantly lower affinity for VAChT than (+)-FBT (**9**) (Ki = 0.22 nM). However, (−)-[^18^F]FBBV (**18**) displayed significantly lower affinity for *σ*_1_ and *σ*_2_ than vesamicol (Ki = 25.8 nM (*σ*_1_) and Ki = 34.5 nM (*σ*_2_)). The brain uptake of (−)-[^18^F]FBBV (**18**) in rats was 0.823, 0.226, 0.124, and 0.095 %ID/g at 5, 30, 60, and 120 min after injection, respectively. (−)-[^18^F]FBBV (**18**) displayed a low brain uptake in rats. The accumulation ratios of the striatum to cortex were 1.39, 1.36, and 1.09 at 30, 60, and 120 min after injection, respectively. The accumulation of (−)-[^18^F]FBBV (**18**) in the rat brain is insufficient for in vivo brain imaging for PET.

#### 2.5.2. (−)-(3-(Hydroxy-1,2,3,4-tetrahydronaphthalen-2-yl)piperidin-4-yl)(4-methoxyphenyl)methanone (**19**)

The affinity (Ki) of (−)-**19** to VAChT, *σ*1, and *σ*2 was 1.6 nM (PC12^A123.7^ cells which express human VAChT), 62.1 nM (Guinea pig brain), and 2,586 nM (rat liver), respectively [79, 80]. However, in the in vitro binding assay, vesamicol as a reference was not assessed simultaneously. The brain uptake of (−)-[^11^C]**19** in rats was 0.560, 0.337, and 0.201 %ID/g at 2, 30, and 60 min after injection, respectively. The accumulation of (−)-[^11^C]**19** in the striatum, cortex, and cerebellum at 30 min after injection was 0.546, 0.331, and 0.317 %ID/g, respectively. The accumulation ratios of the striatum to cortex (ST/CTX) and the striatum to cerebellum (ST/CBL) were 1.65 and 1.72, respectively.

#### 2.5.3. (−)-(1-(3-Hydroxy-1,2,3,4-tetrahydronaphthalen-2-yl)piperidin-4-yl)(4-(methylamino)phenyl)methanone ((−)-TZ659 (**20**))

The affinity (Ki) of (−)-TZ659 (**20**) to VAChT, *σ*_1_, and *σ*_2_ was 0.78 nM (PC12^A123.7^ cells which express human VAChT), 992 nM (Guinea pig brain), and 11443 nM (rat liver), respectively [81, 82]. (−)-TZ659 (**20**) displayed a 3.5-fold higher affinity for VAChT than (−)-FBBV (**16**) and displayed a significantly lower affinity for *σ*_1_ and *σ*_2_ than (−)-FBBV (**20**) [71]. The affinity of vesamicol to VAChT, *σ*_1_, and *σ*_2_ as a reference was not investigated by the same in vitro binding assay method. The accumulation levels of (−)-[^11^C]TZ659 (**20**) in the striatum, cortex, and cerebellum at 30 min after injection were 0.593, 0.262, and 0.157 %ID/g, respectively. The accumulation ratios of the striatum to cortex (ST/CTX) and the striatum to cerebellum (ST/CBL) were 2.26 and 3.78, respectively [81]. PET studies of (−)-[^11^C]TZ659 (**20**) in a male cynomolgus monkey were performed. (−)-[^11^C]TZ659 (**20**) displayed high accumulation in the striatum and low accumulation in the cerebellum [82].

#### 2.5.4. (−)-(1-(8-(2-Fluoroethoxy)-3-hydroxy-1,2,3,4-tetrahydronapthalen-2-yl)piperin-4yl)(4-fluorophenyl)methanone ((−)-VAT (**21**)) [83]

(−)-VAT (**21**) is a vesamicol analog that incorporated the 2-fluoroethoxy group into the 8th position of a tetrahydronapthalene skeleton of (−)-FBBV (**18**). The affinity (Ki) of (−)-VAT (**21**) to VAChT, *σ*_1_, and *σ*_2_ was 0.59 nM (PC12^A123.7^ cells which express human VAChT), >10,000 nM (Guinea pig brain), and >10,000 nM (rat liver), respectively. (−)-VAT (**21**) displayed a 4.6-fold higher affinity for VAChT than FBBV (**18**) and displayed a significantly lower affinity for *σ*_1_ and *σ*_2_ than FBBV (**18**). However, the affinity of vesamicol to VAChT obtained by a different binding assay method was used as a reference. The brain uptake levels of (−)-[^18^F]VAT (**21**) in rats were 0.684, 0.482, 0.425, and 0.409 %ID/g at 5, 30, 60, and 120 min after injection, respectively. The accumulation ratios of the striatum to cortex (ST/CTX) and the striatum to cerebellum (ST/CBL) were about 2.0 and 2.8 at 60 min after injection, respectively. PET studies of (−)-[^18^F]VAT (**21**) in a male cynomolgus monkey were performed. (−)-[^18^F]VAT (**21)** displayed high accumulation in the striatum and low accumulation in the cerebellum.

#### 2.5.5. (−)-(4-((2-Fluoroethyl)amino)phenyl)(1-(3-hydroxy-1,2,3,4-tetrahydronaphthalen-2-yl)piperidin-4-yl)methanone (**22**)

**22** is a vesamicol analog that incorporated the fluoroethyl amino group instead of the methylamino group into (−)-TZ659 (**20**) [84]. The affinity (Ki) of (**22**) to VAChT, *σ*1, and *σ*2 was 0.31 nM (PC12^A123.7^ cells which express human VAChT), 1,870 nM (Guinea pig brain), and 5,480 nM (rat liver), respectively. PET studies of (−)-[^18^F]**22** in a male cynomolgus macaque were performed. (−)-[^18^F]**22** displayed high accumulation in the striatum and low accumulation in the cerebellum.

#### 2.5.6. Summary of Vesamicol Analogs That Incorporated a Carbonyl Group between the B Ring and C Ring of Benzovesamicol

Benzovesamicol analogs that incorporated a carbonyl group displayed low affinity for sigma receptors (*σ*_1_ and *σ*_2_). These compounds (**12** and **16**–**21**) displayed a high selectivity for VAChT over sigma receptors (*σ*_1_ and *σ*_2_). However, because the affinity of vesamicol, as a reference, for VAChT, *σ*_1_, and *σ*_2_ and the affinity of sigma ligands such as pentazocine (*σ*_1_) and DTG (*σ*_2_), as a reference, for *σ*_1_ and *σ*_2_ were not investigated by the same in vitro binding assay method, it is difficult to compare between other vesamicol analogs (**1**–**17**) and these compounds (**18**–**22**) regarding the affinity for VAChT and sigma receptors (*σ*_1_ and *σ*_2_). These compounds (**18**–**22**) displayed low accumulation in the brain against the physicochemical characteristics such as molecular weight (MW = 354–405) and lipophilicity (ALog *D* or Log *P* = 2–3) [71, 81, 83, 84]. Tu et al. reported that the accumulation of (−)-[^11^C]TZ659 (**20**) in the brain increased 2.2-fold by pretreatment with cyclosporine A (CycA), which inhibits a P-glycoprotein related to the drug excretion mechanism, 30 min prior to injection of a radiotracer [83]. On the contrary, 1-(4-[^18^F]fluorobenzyl)-4-[(tetrahydrofuran-2yl)methyl]piperizine, which displayed a high brain uptake as a *σ*_1_ receptor ligand, was unaffected by pretreatment with CycA [81]. These results showed that (−)-[^11^C]TZ659 (**20**) was a substrate of CycA with low affinity, and the low accumulation of (−)-[^11^C]TZ659 (**20**) in the brain was caused by CycA into the blood-brain barrier (BBB).

## 3. Discussion

### 3.1. Cholinergic Nerve Systems

The cholinergic system includes the following three main cholinergic pathways [85–90]: (1) numerous cholinergic neurons in the basal forebrain, which supply cholinergic projections throughout the cerebral cortex, the forebrain limbic structures, the diagonal band nucleus, the amygdala, and the hippocampus, (2) the mesopontine regions including the laterodorsal tegmental nucleus and the pedunculopontine nucleus, which project to the forebrain, the thalamus, the hypothalamus, the cerebellar nucleus, and the brainstem, and (3) populations of cholinergic interneurons in the striatum. Cholinergic neurons in the basal forebrain and the mesopontine regions are closely associated with cognition, learning, and memory functions. Cholinergic neurons in the striatum do not project beyond the borders of the striatum. It is important to investigate the change of the cholinergic nerve system in the cerebral cortex, the forebrain limbic structures, the diagonal nucleus, the amygdala, the hippocampus, the thalamus, the hypothalamus, and the cerebellar nucleus in comparison with the striatum in order to diagnose AD early by a VAChT imaging agent.

### 3.2. Binding Affinity of Vesamicol Analogs for VAChT, *σ*_1_, and *σ*_2_

It is difficult to compare between vesamicol analogs (**1**–**22**) because the affinity of vesamicol, as a reference, to VAChT, *σ*_1_, and *σ*_2_ was not investigated by the same in vitro binding assay method. In this report, the affinity of vesamicol analogs (**1**–**22**) to VAChT, *σ*_1_, and *σ*_2_ was compared with that of vesamicol as a reference (Table 1). (±)-MIBT (**8**) displayed a high affinity and high selectivity for VAChT using *Torpedo californica* as tissue preparations. However, Custers et al. reported that (+)-MIBT (**8**), FBT (**7**), and oIV (**10**) displayed higher affinity for *σ*_1_ and *σ*_2_ than vesamicol by the same in vitro binding assay method. OIDV (**13**), OBDV (**14**), and 3′-IBVM (**12**) displayed higher affinity for VAChT and lower affinity for *σ*_1_ and *σ*_2_ than vesamicol. (−)-FBBV (**18**) displayed the lowest affinity for *σ*_1_ and *σ*_2_. Vesamicol analogs (**19**–**22**), which incorporated a carbonyl group between the B ring and C ring, and (−)-FBBV (**18**) are expected to display low affinity for *σ*_1_ and *σ*_2_. Finally, to investigate the affinity of vesamicol analogs to VAChT, *σ*_1_, and *σ*_2_, it is necessary that vesamicol and novel vesamicol analogs for VAChT, *σ*_1_, and *σ*_2_ were assayed by the same in vitro binding assay simultaneously.

### 3.3. Distribution of VAChT in Cholinergic Neurons

The nerve terminal consists of presynaptic and postsynaptic neurons. The regional distribution of VAChT situated at presynaptic cholinergic nerve terminals is similar to that of acetylcholine receptors situated at the postsynapses of cholinergic nerve terminals. Muscarinic acetylcholine (M_1_–M_5_) receptors (mAChR (M_1_–M_5_)) are found in high density in the cerebral cortex, striatum, diagonal band, hippocampus, amygdala, anterior and intralaminar nuclei of the thalamus, granule and purkinje cell layers of the cerebellum, and motor nuclei of the cranial nerves [91–93]. The nicotinic receptor is widely distributed in the anteroventral nucleus of the thalamus [94]. Therefore, VAChT-rich presynaptic cholinergic nerve terminals were thought to be widely distributed in various brain regions, including the cerebral cortex, striatum, diagonal band, hippocampus, thalamus, amygdaloid nucleus, cerebellum, and nuclei of cranial nerves. The mAChR concentrations in the striatum are approximately 1.67-fold higher than those in the cerebral cortex.  Table 2 shows the accumulation of vesamicol analogs (**1**–**22**) in the striatum and cortex and the ratio of the striatum to cortex (ST/CTX) for radiolabeled vesamicol analogs. CNS radioligands need to accumulate in the brain through the BBB. The CNS radioligands are required to have 2-3 as the value of the log of the octanol-water partition coefficient (Log *P*) and be less than 500 as molecular weight (MW) as their chemical characteristics to penetrate the BBB [95, 96]. The vesamicol analogs (**1**–**22**) except for (−)-VAT (**21)** displayed physicochemical properties such as molecular weight (MW = 272–474) and lipophilicity (Log *P* = 2-3). (−)-oIV (**10)** and (−)-OMV (**11)** displayed high brain uptake in rat. However, other vesamicol analogs displayed low brain uptake in rats or mice against the physicochemical properties. The low brain uptake of vesamicol analogs except for (−)-oIV (**10)** and (−)-OMV (**11)** may be caused by excretion of vesamicol analogs from brain by a P-glycoprotein related to the drug excretion mechanism or the high binding affinity for serum protein. The accumulation of (−)-[^11^C]TZ659 (**20**) in the brain increased 2.2-fold by pretreatment with cyclosporine A (CycA), which inhibits a P-glycoprotein related to the drug excretion mechanism, 30 min prior to injection of a radiotracer. The vesamicol analogs except for (−)-oIV (**10)**, (−)-OMV (**11**), and (−)-OIDV (**13**) showed a ratio of the striatum to cortex (ST/CTX) more than 1.1. As mentioned above, the VAChT-rich region was widely distributed in the various regions of the brain. These VAChT imaging agents will distribute in the cerebral cortex, the hippocampus, the thalamus, the hypothalamus, and the cerebellum besides the striatum. However, several vesamicol analogs showed the high concentration in the striatum and the low concentration in the cerebral cortex and the cerebellum.

### 3.4. The Influence of Enantioselectivity of Vesamicol Analogs on the In Vitro Binding Affinity for VAChT and In Vivo Brain Uptake

The influence of enantioselectivity of the vesamicol analogs FEOBV (**2**) [45], MIBT (**8**) [55], FBT (**9**) [60], oIV (**10**) [62], OMV (**11**) [63], OIDV (**13**) [67], FBBV (**18**) [78], **19** [81], TZ659 (**20**) [82], VAT (**21**) [83], and **22** [84] on the binding affinity for VAChT, *σ*_1_, and *σ*_2_ receptors was reported. The investigated vesmicol analogs except for MIBT (**8**) and FBT (**9**) showed that (−)-enantiomers had about 3- to 29-fold higher binding affinity for VAChT than (+)-enantiomers. MIBT (**8**) and FBT (**9**) showed that (+)-enantiomers had about 40- to 70-fold higher binding affinity for VAChT than (−)-enantiomers. On the contrary, several investigated vesamicol analogs such as oIV (**10**), OMV (**11**), and FBBV (**18**) showed that (−)-enantiomers had more than 3-fold lower binding affinity for *σ*_1_ receptors than (+)-enantiomers (Table 3). The accumulation and retention of the investigated vesamicol analogs except for MIBT (**8**) in brain uptake showed that (−)-enantiomers had higher accumulation and retention in the brain than (+)-enantiomers. The accumulation ratios of the striatum to cortex (ST/CTX) of the investigated vesamicol analogs except for [^18^F]FBT (**9**) showed that (−)-enantiomers were superior to (+)-enantiomers (Table 4).

### 3.5. The Structure-Activity Relationship of Radioligands for VAChT Imaging

In in vitro characterization, such as the affinity and selectivity of radioligands for VAChT, (−)-enantiomers of vesamicol analogs based on benzovesamicol ((−)-FEOBV (**2**) and (−)-FBBV (**18**)) are superior to other vesamicol analogs (Table 1). However, benzovesamicol analogs showed low brain uptake in the rat and mouse. On the contrary, in in vivo characterization, such as brain uptake of radioligands, vesamicol analogs that incorporated a radionuclide into the C ring of vesamicol ((−)-oIV (**10**) and (−)-OMV (**11**)) were superior to other vesamicol analogs (Table 2). However, (−)-oIV (**10**) and (−)-OMV (**11**) showed low selectivity for VAChT in vitro and in vivo. Considering abovementioned results, the in vivo characterization of radioligands for VAChT will improve by minimizing the molecular weight of the ligand, and in vitro characterization of radioligands for VAChT will improve by incorporating a carbonyl group between the B ring and C ring of vesamicol analogs. We are interested in vesamicol analogs incorporating three elements such as a carbonyl group between the B ring and C ring, a 4- to 7-membered alicyclic ring as A ring, and a radionuclide in the C ring, together.

## 4. Conclusion

Many vesamicol analogs were investigated as an VAChT imaging agent. In this report, 5 types of vesamicol analogs were investigated: (1) vesamicol analogs based on benzovesamicol, (2) vesamicol analogs based on trozamicol, (3) vesamicol analogs that incorporated a radionuclide into the C ring of vesamicol, (4) vesamicol analogs that incorporated alicyclic groups into the A ring of vesamicol, and (5) vesamicol analogs that incorporated a carbonyl group between the B ring and C ring of benzovesamicol. All vesamicol analogs (**1**–**22**) are insufficient as an VAChT imaging agent for early diagnosis of Alzheimer's disease. This is because the vesamicol analogs with a high affinity and a high selectivity for VAChT showed low brain uptake, and vesamicol analogs with a high brain uptake showed high affinity for sigma receptors and low selectivity for VAChT. Considering the relationship between the cholinergic nerve system and AD, the development of a VAChT imaging agent is important. It is necessary that the suitable radioligand for VAChT imaging shows a high affinity and high selectivity for VAChT in vitro and in vivo and shows the high accumulation of the regional brain in accordance with the concentration distribution of VAChT in the brain. Furthermore, the ideal radioligand for VAChT imaging will require the fast blood clearance and the resistance to cleavage of the radioligand as described by Giboureau et al. previously. In the future, the suitable cholinergic neuronal degeneration imaging is thought to be found by comparing these three imaging: ChT imaging, VAChT imaging, and ChT imaging, and putting them together. It is necessary to further the development of a radioactive imaging agent for choline transporter (ChT) and choline acetyl transferase (ChAT). Finally, the ideal AD imaging is thought to be obtained by putting amyloid imaging, tau imaging, and cholinergic neuronal imaging together.

## Figures and Tables

**Table 1:** Binding affinity of vesamicol analogs for VAChT, *σ*_1_, and *σ*_2_.

	The ratio of vesamicol analogs (**1**–**22**) to vesamicol for Ki value			Tissue preparations for VAChT
	VAChT	*σ*_1_	*σ*_2_	
Vesamicol	1	1	1	—
Benzovesamicol (BV)	0.56	0.80	0.86	Rat brain membranes
Decalinvesamicol (DV)	0.50	2.75	0.31	Rat brain membranes
(−)-IBVM (**1**)^*∗*^	—	—	—	—
(−)-FEOBV (**2**)^*∗∗*^ [45]	0.41	10.25	—	rVAChT-PC12 cell
(−)-MABV (**3**) [48]	—	—	—	—
(−)-NEFA (**4**) [49]	0.32	—	—	*Torpedo californica*
(**5**) [51]	1.17	22.84	11.38	rVAChT-PC12 cell
(**6**) [53]	0.49	3.19	0.54	rVAChT-PC12 cell
(**7**) [53]	0.14	0.44	2.30	rVAChT-PC12 cell
MIBT (**8**) [55]	0.065	3.54	5.59	*Torpedo californica*
(+)-MIBT (**8**) [55]	0.11	0.13	0.51	Rat brain membranes
FBT (**9**) [55]	0.25	0.04	0.66	Rat brain membranes
(+)-FBT (**9**) [60]	0.22	0.84	1.04	*Torpedo californica*
oIV(**10**) [62]	0.42	0.077	0.83	Rat brain membranes
(−)-oIV (**10**) [62]	1.15	0.83	1.32	Rat brain membranes
(−)-OMV (**11**) [63]	1.52	0.46	0.77	Rat brain membranes
3′-IBVM (**12**) [64]	0.21	5.75	1.37	*Torpedo californica*
OIDV (**13**) [65]	0.61	10.94	1.37	Rat brain membranes
(−)-OIDV (**13**) [66]	0.95	8.66	0.86	Rat brain membranes
OBDV (**14**) [67]	0.41	6.82	1.59	Rat brain membranes
OMDV (**15**) [68]	0.56	3.38	0.31	Rat brain membranes
FAMV (**16**) [69]	—	—	—	rVAChT-PC12 cell
FBMV (**17**) [70]	—	—	—	rVAChT-PC12 cell
(−)-FBBV (**18**) [71]	0.51	13.90	9.25	*Torpedo californica*
(−)**-19**^*∗∗*^	—	—	—	rVAChT-PC12 cell
(−)-TZ659 (**20**)^*∗∗*^	—	—	—	rVAChT-PC12 cell
(−)-VAT (**21**)^*∗∗*^	—	—	—	rVAChT-PC12 cell
(−)-**22**^*∗∗*^	—	—	—	rVAChT-PC12 cell

^*∗*^The affinity of these vesamicol analogs for VAChT, *σ*1, and *σ*2 had not been reported. ^*∗∗*^The affinity of vesamicol, as a reference, for VAChT, *σ*1, and *σ*2 was not investigated by the same in vitro binding assay method.

**Table 2:** Accumulation of vesamicol analogs (**1**–**22**) in the striatum and cortex and the ratio of the striatum to cortex (ST/CTX) for radiolabeled vesamicol analogs accumulation.

	%ID/g		ST/CTX	Animal	Molecular weight	Partition coefficient
	Cortex	Striatum				
[^3^H]vesamicol [74]	0.68 (60 min)	0.74 (60 min)	1.09 (60 min)	Rats	259	1.40^*∗*^
Benzovesamicol	—	—	—	—	307	2.44^*∗*^
[^123^I]IBVM (**1**) [37, 42]	2.56 (240 min)	6.26 (240 min)	2.45 (240 min)	Mice	429	—
	0.3 (120 min)	0.53 (120 min)	1.77 (120 min)	Rats		—
(−)-[^18^F]FEOBV (**2**) [46]	4.65 (45 min)	8.09 (45 min)	1.74 (45 min)	Mice	368	—
(−)-[^11^C]MABV (**3**) [48]	3.99 (45 min)	8.1 (45 min)	2.6 (75 min)	Mice	336	—
(−)-[^18^F]NEFA (**4**) [49]	—	—	2.5–3 (50 min)	Monkeys	406	—
(+)-[^125^I]MIBT (**8**) [56]	0.40 (120 min)	0.67 120 min)	1.68 (120 min)	Rats	474	—
(+)-[^18^F]FBT (**9**) [72]	0.58 (60 min)	0.74 (60 min)	1.28 (60 min)	Rats	367	1.99^*∗*^
(−)-[^125^I]oIV (**10**) [74]	1.55 (60 min)	1.57 (60 min)	1.01 (60 min)	Rats	383	2.15^*∗∗*^
(−)-[^11^C]OMV (**11**) [75]	1.16 (30 min)	1.07 (30 min)	0.92 (30 min)	Rats	272	—
3′-[^125^I]IBVM (**12**) [64]	0.38 (60 min)	0.61 (60 min)	1.61 (60 min)	Rats	431	—
(−)-[^123^I]OIDV (**13**) [66]	0.62 (30 min)	0.63 (30 min)	1.02 (30 min)	Rats	435	2.46^*∗∗*^
[^77^Br]OBDV (**14**) [67]	0.52 (30 min)	0.59 (30 min)	1.13 (30 min)	Rats	389	2.93^*∗∗*^
[^11^C]OMDV (**15**) [68]	0.57 (60 min)	0.72 (60 min)	1.26 (60 min)	Rats	326	2.37^*∗∗*^
[^18^F]FAMV (**16**) [69]	—	—	4.5 (60 min)	Rats	361	1.88
[^18^F]FBMV (**17**) [70]	0.09 (60 min)	0.17 (60 min)	3.4 (60 min)	Rats	423	2.10
(−)-[^18^F]FBBV (**18**) [71]	—	—	1.36 (60 min)	Rats	352	2.99^*∗*^
(−)-[^11^C]**19** [80]	0.331 (30 min)	0.546 (30 min)	1.65 (30 min)	Rats	364	—
(−)-[^11^C]TZ659 (**20**) [81]	0.262 (30 min)	0.593 (30 min)	2.26 (30 min)	Rats	363	2.61^*∗*^
(−)-[^18^F]VAT (**21**) [83]	—	—	4.19 (60 min)	Rats	414	3.45^*∗*^
(−)-[^18^F]**22** [84]	—	—	1.76 (60 min)	Monkeys	395	2.91^*∗*^

^*∗*^Calculated value (Log *P*) at pH 7.4 by ACD/Laboratories, version 7.0 (Advanced Chemistry Development, Inc., Canada). ^*∗∗*^Log *P*_o/w_ = log_10_ (radioactivity in *n*-octanol/radioactivity in 0.1 M phosphate buffer (pH = 7.4)).

**Table 3:** The ratio of binding affinity (Ki) of enantiomers of vesamicol analogs for VAChT, *σ*_1_, and *σ*_2_ receptors ((−)-body/(+)-body).

	The ratio of binding affinity (Ki) of enantiomers of vesamicol analogs for VAChT, *σ*1, and *σ*2 receptors ((−)-body/(+)-body)			Tissue preparations for VAChT
	VAChT	*σ*_1_	*σ*_2_	
Vesamicol [55]	0.034	4.88	1.12	Rat brain membranes
FEOBV (**2**) [45]	0.344	0.78	—	rVAChT-PC12 cell
MIBT (**8**) [55]	39.6	0.23	1.45	Rat brain membranes
FBT (**9**) [60]	72.7	1.03	3.05	*Torpedo californica*
oIV (**10**) [62]	0.063	4.29	2.80	Rat brain membranes
OMV (**11**) [63]	0.30	3.15	1.22	Rat brain membranes
OIDV (**13**) [66]	0.28	0.53	0.59	Rat brain membranes
FBBV (**18**) [71]	0.038	3.54	1.15	*Torpedo californica*
**19** ^*∗∗*^ [80]	0.047	0.47	1.74	rVAChT-PC12 cell
TZ659 (**20**) [81]	0.041	0.85	1.47	rVAChT-PC12 cell
VAT (**21**) [71]	0.045	1.0	>1.28	rVAChT-PC12 cell
**22** [84]	0.13	1.50	0.55	rVAChT-PC12 cell

**Table 4:** Accumulation of enantiomers of vesamicol analogs (**1**–**18**) in the striatum and cortex and the ratio of the striatum to cortex (ST/CTX) for enantiomers of radiolabeled vesamicol analogs.

	%ID/g			ST/CTX	Animal
	Cortex	Striatum	Whole brain		
(−)-[^125^I]IBVM (**1**) [36]	2.56 (240 min)	6.26 (240 min)	—	2.44	Mouse
(+)-[^125^I]IBVM (**1**) [36]	0.12 (240 min)	0.26 (240 min)	—	2.17	Mouse
(−)-[^18^F]FEOBV (**2**)^∗^ [45]	4.5 (180 min)	9.0 (180 min)	—	1.74	Mouse
(+)-[^18^F]FEOBV (**2**)^∗^ [45]	2.0 (180 min)	1.9 (45 min)	—	0.95	Mouse
(−)-[^11^C]MABV (**3**)	3.99 (45 min)	8.1 (45 min)	—	2.03	Mouse
(+)-[^11^C]MABV (**3**)	0.91 (45 min)	1.23 (45 min)	—	1.35	Mouse
(−)-[^125^I]MIBT (**8**) [52]	—	—	0.27 (60 min)	—	Rat
(+)-[^125^I]MIBT (**8**) [52]	—	—	0.83 (60 min)	—	Rat
(−)-[^18^F]FBT (**9**) [57]	1.18 (60 min)	1.03 (60 min)	1.05 (60 min)	0.87	Rat
(+)-[^18^F]FBT (**9**) [57]	0.58 (60 min)	0.74 (60 min)	0.59 (60 min)	1.28	Rat
(−)-[^123^I]OIDV (**13**) [63]	0.62 (30 min)	0.63 (30 min)	—	1.02	Rat
(+)-[^123^I]OIDV (**13**) [63]	0.46 (30 min)	0.45 (30 min)	—	0.98	Rat
(−)-[^18^F]FBBV (**18**) [77]	—	—	0.226 (30 min)	—	Rat
(+)-[^18^F]FBBV (**18**) [77]	—	—	0.071 (30 min)	—	Rat

^*∗*^The value that was read from a graph was used.
